# Situational judgment test as an additional tool in a medical admission test: an observational investigation

**DOI:** 10.1186/s13104-015-1033-z

**Published:** 2015-03-14

**Authors:** Marion Luschin-Ebengreuth, Hans P Dimai, Daniel Ithaler, Heide M Neges, Gilbert Reibnegger

**Affiliations:** Vice-Rector’s Office for Teaching and Studies, Medical University Graz, Auenbruggerplatz 2/4, Graz, 8036 Austria; Organizational Unit for Studies and Teaching, Medical University Graz, Harrachgasse 21/6, Graz, 8010 Austria; Institute of Physiological Chemistry, Center for Physiological Medicine, Medical University Graz, Harrachgasse 21/II, Graz, 8010 Austria

**Keywords:** Situational judgment test, Medical University, Medical admission test

## Abstract

**Background:**

In the framework of medical university admission procedures the assessment of non-cognitive abilities is increasingly demanded. As tool for assessing personal qualities or the ability to handle theoretical social constructs in complex situations, the Situational Judgment Test (SJT), among other measurement instruments, is discussed in the literature. This study focuses on the development and the results of the SJT as part of the admission test for the study of human medicine and dentistry at one medical university in Austria.

**Methods:**

Observational investigation focusing on the results of the SJT. 4741 applicants were included in the study. To yield comparable results for the different test parts, “relative scores” for each test part were calculated. Performance differences between women and men in the various test parts are analyzed using effect sizes based on comparison of mean values (Cohen’s d). The associations between the relative scores achieved in the various test parts were assessed by computing pairwise linear correlation coefficients between all test parts and visualized by bivariate scatterplots.

**Results:**

Among successful candidates, men consistently outperform women. Men perform better in physics and mathematics. Women perform better in the SJT part. The least discriminatory test part was the SJT. A strong correlation between biology and chemistry and moderate correlations between the other test parts except SJT is obvious. The relative scores are not symmetrically distributed.

**Conclusions:**

The cognitive loading of the performed SJTs points to the low correlation between the SJTs and cognitive abilities. Adding the SJT part into the admission test, in order to cover more than only knowledge and understanding of natural sciences among the applicants has been quite successful.

## Background

Medical university admission tests/admission procedures fulfill the demand of selecting potential students and are used as predictors for the educational success of the college applicants. Admission tests thus (i) have to guarantee the fair and reproducible allocation of limited university places to a preferably diverse future student population [1,2], (ii) should select those applicants who, with the greatest probability, develop – hard to define - abilities and characteristics that are expected from future physicians [3-5] and, (iii) should identify those applicants who show the greatest probability of finishing the course of study [3,6,7]. In addition to the assessment of cognitive abilities, the assessment of non-cognitive abilities is increasingly demanded [8]. In this context various methods for determining “soft skills”, (inter) personal skills or the ability to handle theoretical social constructs (e.g., health/sickness, ethnicity, gender) in complex situations were evaluated [9]. As instruments for assessing personal qualities, different tools are discussed in the literature [10]:

- the interview, with no attested positive predictive validity for medical school applicants [11] and disputable reliability [5,12];
- psychometric assessments (as for example, the Personal Qualities Assessment (PQA)) are – assuming further development – assigned definite potential [4,12];
- the Multiple Mini Interview (MMI) which, in studies, among other things, is attested a statistically significant, predictive validity for the future performance of participants [8,11,12];
- letters of recommendation as well as personal and autobiographical statements – whose reliability or predictive validity to date was not yet confirmed [12].

A further assessment instrument is the Situational Judgment Test (SJT) [13,14]. The SJT assesses – as McDaniel et al. [13] summarize in their meta-analysis – a plurality of constructs [13,15]. Following this result, O’Connell et al. [16] recommend to interpret SJTs best as measurement methods and not measures of a single construct [16]. At any rate, the SJT is attested validity as a predictor for future job performance [17] and – assuming that relevant work-related situations are described – face and content validity [17,18].

As the only one of the three Austrian medical universities, the Medical University of Graz has amended its admission process (cognitive testing with the subsections biology, chemistry, physics and mathematics as well as the testing of text comprehension) by including a written Situational Judgment Test (SJT) in the year 2010 [19-21].

## Method

### Study population

This study is an observational investigation focusing on the results of the situational judgment test (SJT) as part of the admission test for the study of human medicine and dentistry at the Medical University of Graz, obtained in the academic years 2010/11, 2011/12 and 2012/13. Over the three years, there were 4741 applicants, all of whom were included in the study. (The distributions of applicants for the time period investigated are depicted in Table 1).

**Table 1:** **Distributions of applicants as well as of successful applicants according to sex and nationality in three consecutive academic years**

**Admission test**	**Applicants from**	**Total**	**Women**		**Men**		**Successful applicants from**	**Total**	**Women**		**Men**	
			**Number**	**%**	**Number**	**%**			**Number**	**%**	**Number**	**%**
**2010**	Austria	1029	576	55.98	453	44.02	Austria	274	122	44.53	152	55.47
	European Union	298	149	50.00	149	50.00	European Union	74	37	50.00	37	50.00
	Other nationalities*	26	7	26.92	19	73.08	Other nationalities	18	4	22.22	14	77.78
	All nationalities	1353	732	54.10	621	45.90	All nationalities	366	163	44.54	203	55.46
**2011**	Austria	1190	690	57.98	500	42.02	Austria	281	142	50.53	139	49.47
	European Union	493	268	54.36	225	45.64	European Union	76	34	44.74	42	55.26
	Other nationalities	19	10	52.63	9	47.37	Other nationalities	9	5	55.56	4	44.44
	All nationalities	1702	968	56.87	734	43.13	All nationalities	366	181	49.45	196	50.55
**2012**	Austria	1164	661	56.79	503	43.21	Austria	284	126	44.37	158	55.63
	European Union	510	288	56.47	222	43.53	European Union	76	32	42.11	44	57.89
	Other nationalities	12	5	41.67	7	58.33	Other nationalities	5	2	40.00	3	60.00
	All nationalities	1686	954	56.58	732	43.42	All nationalities	365	160	43.84	205	56.16
**2010 - 2012**	Austria	3383	1927	56.96	1456	43.04	Austria	839	390	46.48	449	53.52
	European Union	1301	705	54.19	596	45.81	European Union	226	103	45.58	123	54.42
	Other nationalities	57	22	38.60	35	61.40	Other nationalities	32	11	34.38	21	65.63
	All nationalities	4741	2654	55.98	2087	44.02	All nationalities	1097	504	45.94	593	54.06

### Admission examination measures: cognitive test & situational judgment test

#### Cognitive test

The cognitive test, as applied in the academic years investigated, is based on secondary school level knowledge in biology, chemistry, physics and mathematics, and additionally contains a text comprehension test part. (The number of items in the individual subareas is depicted in Table 2). These five different test disciplines (biology, chemistry, physics, mathematics, and text comprehension) and the SJT (the sixth test discipline) are designed “test parts”. All test parts are uniformly done in the format of a written multiple choice test. Specifically, for each test item there are four distractors, one of which represents the correct answer. For correct answers, the applicants receive positive scores of 2 (5 in the case of text comprehension part) in dependence on the test part; for wrong answers a negative score of −1 is counted. The rationale behind this scoring is twofold: first, guessing should be discouraged. Second, in medicine a critical self-evaluation of one’s knowledge is imperative, and thus, applicants should be encouraged to critically self-assess their knowledge before answering a test item. Leaving out an item without choosing one of the four distractors leads to a score of 0 for this item. For the determination of the ranking of the applicants – and hence, for the decision whether or not an applicant was admitted, − the scores for each item are summed up to give a total score. Due to the different number of items in the various test parts, there is an implicit weight given to each of these parts.

**Table 2:** **Mean relative scores showing the performance of women and men in the various test parts**

**Academic year**		**2010/11**				**2011/12**				**2012/13**		
	**N** ^**§**^	**Relative scores**			**N**	**Relative scores**			**N**	**Relative scores**		
**Tes part**		Women*	**Men**	**Cohen’s d** ^**#**^		**Women**	**Men**	**Cohen’s d**		**Women**	**Men**	**Cohen’s d**
**Biology**	90	.526	.558	.21	50	.546	.572	.14	50	.544	.577	.20
		(.153)	(.149)	(.11 – .32)		(.178)	(.182)	(.05 – .24)		(.165)	(.171)	(.10 – .29)
**Chemistry**	30	.519	.556	.22	30	.540	.582	.24	30	.577	.640	.33
		(.164)	(.173)	(.11 – .33)		(.173)	(.174)	(.15 – .34)		(.192)	(.192)	(.23 – .43)
**Physics**	20	.410	.465	.40	20	.443	.516	.47	20	.446	.521	.45
		(.128)	(.143)	(.30 – .51)		(.148)	(.168)	(.37 – .57)		(.158)	(.177)	(.36 – .55)
**Mathematics**	20	.520	.563	.27	20	.530	.606	.46	20	.522	.600	.48
		(.148)	(.167)	(.16 – .38)		(.159)	(.171)	(.36 – .56)		(.154)	(.173)	(.38 – .58)
**Text comprehension**	20	.631	.644	.08	34	.640	.664 (.157)	.15	30	.663	.690	.18
		(.157)	(.155)	(−.02 – .19)		(.152)		(.05 – .25)		(.153)	(.152)	(.08 – .28)
**SJT**	20	.857	.843	-.14	30	.785	.761	-.19	30	.868	.849	-.22
		(.095)	(.102)	(−.25 – -.04)		(.130)	(.133)	(−.28 – -.09)		(.083)	(.088)	(−.32 – -.12)

^§^Number of items.

*Values are mean relative scores and standard deviation in parentheses.

^#^Values are Cohen’s d and 95% confidence interval in parentheses.

#### Situational judgment test

The development of the SJT items proceeded in four phases, using lecturers/professors and advanced students [14,22].

Phase 1: In the framework of a seminar at the Medical University Graz (MUG), students with a minimum of study experience of 4–6 semesters were given the task to describe critical situations that were experienced in a medical context (in the role of patient, family member, student, etc.) as particularly appropriate or particularly inappropriate. The experienced patterns of action were discussed in small groups and additional possible courses of action were developed. The situations described by the students were then presented to a core team of experts, who grouped and selected representative scenarios and adapted the possible routes of action according to form, length and style, in order to create the actual test items. The following set of criteria was used:

- the comprehensible context/the possible reference to basic statements of the bio-psycho-social model (information regarding the bio-psycho-social model was made available to all college applicants with a notice regarding its relevance for the test),
- the degree of difficulty (no medical (pre)-knowledge is necessary for responding) and
- logical coherence.

Phase 2: Critical evaluation and extension of possible courses of action of the situational descriptions – included in the further process – by professors and lecturers.

Phase 3: Evaluation of the courses of action by the steering committee (professors/lecturers/psychologists) and discussion about or determination of the sequence of potential courses of action by the steering committee together with the core team.

Phase 4: Performance of a pre-test, again modification of the SJT items, taking into account the results of the pre-test. Final revision and approval [23].

#### Perceptions of the admission examination by the examinees

In 2010, after having completed the admission test, the applicants were invited to provide an evaluation of certain aspects of the procedure. For each part of the admission test, they were asked – among other questions – for their subjective judgment of the difficulty as well as of the importance within the admission test and the importance for their prospective future career in medicine. The candidates were given the opportunity to provide their rating on a 6-point scale (1 = not difficult at all, 6 = very difficult/1 = not meaningful at all, 6 = very meaningful). All data were made anonymous in order to eliminate any retracing.

### Statistical analyses

For each test item, the index of discrimination describing the correlation of that index with the total test is computed. These indices of discrimination are then aggregated for the knowledge test (combined results on biology, chemistry, physics and mathematics), text comprehension test and SJT, separately for each year.

For proper statistical analyses of the results of the various test parts, we take into account the fact that not only the absolute numbers of items are different for each test part, but these numbers also vary from one year to the next (in Table 2, these item numbers per test part and year are explicitly stated). In order to compensate for these variations and to yield comparable results for the different test parts, we calculate “relative scores” for each test part using the following formula:

$$ relative\  score=\frac{score- minimum}{maximum- minimum}. $$

Here, “*score*” is the absolute score of an applicant in a chosen test part, “*minimum*” represents the worst case of answering all items of a test part wrongly, and “*maximum*” denotes the best case of answering all items of a test part correctly. To give an example, suppose an applicant with a biology score of 45. In the respective admission test, suppose there are 90 biology items with possible scores of −1/0/+2, if the answer was false/no answer/correct. In this case, *minimum* = − 90 and *aximum* = 180. The applicant thus has a

$$ relative\  score=\frac{45-\left(-90\right)}{180-\left(-90\right)}=\frac{135}{270}=0.50. $$

Computing relative scores this way ensures that they can range from 0.0 (all items of a test part falsely answered) to 1.0 (all items of a test part correctly answered). (Other normalizing schemes like z-scoring would have been possible; qualitative aspects of the results and conclusions probably would remain basically unchanged).

Basic statistical analyses of these relative scores are performed using the usual descriptive statistical techniques as well as correlation analysis. Performance differences between women and men in the various test parts are analyzed using effect sizes based on comparison of mean values (Cohen’s d) because due to the high frequency of observations even very small differences of mean values become statistically significant in terms of usually employed P-values. Cohen’s d values are generally interpreted as follows: *d* ≤ 0.2 indicates a weak effect, *d* > 0.5 indicates a strong effect, and 0.2 < *d* ≤ 0.5, a moderate effect.

The associations between the relative scores achieved in the various test parts were assessed by computing pairwise linear correlation coefficients between all test parts and visualized by bivariate scatterplots.

All statistical analyses are performed using STATA 13 software (StataCorp. LP, College Station, TX, USA).

### Ethics statement

The authors gathered anonymized data from a data set that is routinely collected about medical students’ admission, dropout, and graduation dates and examination history, as required by the Austrian Federal Ministry of Science and Research. Because the data were anonymous and no data beyond those required by law were collected for this study, the Medical University of Graz’s ethical approval committee did not require approval for this study.

## Results and discussion

### Basic data

For the academic years 2010/11 to 2012/13, Table 1 shows basic data on the admission tests at the Medical University of Graz. As already described in an earlier publication [24], there are consistently more women than men among the applicants. This corresponds extensively with the communicated data on admission processes for Europe. Tiffin et al. [25] describe, for example, that for the UK, women – in relation to the UK population – are over-represented in medical school intakes [25]. In contrast to this, the data from North America indicate a decrease in female applicants [26].

### Sex effects

Table 2 shows the relative scores obtained by women and men in the different test parts as well as the effect size of sex. As can be seen from the mean values of the relative scores, among the natural science parts, physics is the most difficult test part (with the smallest relative scores), while biology, chemistry and mathematics present similar difficulties to the test applicants. Men perform considerably better in physics and mathematics: one result that is confirmed by all public medical universities in Austria [27,28] and discussed internationally, e.g., for physics and biology [2,25,29]. In the literature, stereotyping, different risk behavior in men and women, the factor time or testing anxiety, among other things, are listed as reasons for the gender gap in high stakes tests [24,29]. While in text comprehension men still perform slightly better than women, the reverse is true in SJT; here the negative values of Cohen’s d indicate consistent better performances of women with weak to moderate effect size. The 95% confidence intervals of Cohen’s d show that the observed effect t sizes are significantly different from zero in all cases, with the single exception of text comprehension in 2010/11; here, the confidence interval contains zero.

### Indices of discrimination of the test parts

Table 3 indicates, that in each year studied, the highest mean indices of discrimination were found for the knowledge test part (consisting of biology, chemistry, physics and mathematics), followed by text comprehension, and the least discriminatory test part was, with the exception of 2011, the SJT. The low answer variance for less difficult tasks – in the present case, the questions in the framework of the SJT – influences the mean indices of discrimination. As a further factor that influences the discriminatory power and, ultimately, the validity of, e.g., SJT results, the positioning of the SJT in the whole test is discussed in the literature [30,31]. In this context, Marentette et al. [31] describe construct-irrelevant order effects which occur when longer SJT items and SJT items presented in written form have to be answered at the end of an admission process [31]. Nevertheless, in any case all single test indices of any of the test parts were positive, indicating that participants with higher abilities on average performed better on each single test item.

**Table 3:** **Mean item discrimination indices of the test parts, grouped per year of admission test**

**Year**	**2010**	**2011**	**2012**
**Test part**			
Knowledge test*	0.306	0.342	0.349
**Text compr**	0.238	0.271	0.276
**SJT**	0.196	0.311	0.176

*“Knowledge test” represents the combination of biology, chemistry, physics and mathematics.

### Correlation analyses

Table 4 reports, for each year separately, the pairwise linear correlation coefficients between the relative scores of the various test parts. While due to the large numbers of subjects included, all correlation coefficients are significantly different from zero, there are considerable differences: the highest correlation coefficients are invariably seen between biology and chemistry results. In general, the four natural science scores show relatively strong mutual correlations. Text comprehension is moderately strongly correlated with all other variables, including SJT, but the latter with all other variables except text comprehension shows very weak correlations. This result appears in front of the background that Situational Judgment Inventories measure constructs that are not exclusively identical with cognitive ability, not a big surprise [32]. As possible explanation one could use, among other things, the instruction type (behavioral tendency response instructions) of the performed SJTs. As McDaniel et al. [15] record, in the framework of a “typical performance test” (among other things, SJT with behavioral tendency response instructions), in contrast to “maximal performance tests” (among other things, knowledge test), lower cognitive correlates are to be expected [13,15].

**Table 4:** **Pairwise linear correlation coefficients between relative scores on the various text parts, sorted by year of admission test** *

**a) Admission test 2010 (N = 1353)**					
**Test part**	**Biology**	**Chemistry**	**Physics**	**Mathematics**	**Text comp.**
**Chemistry**	0.732				
**Physics**	0.523	0.586			
**Mathematics**	0.243	0.318	0.463		
**Text comp.**	0.445	0.407	0.354	0.379	
**SJT**	0.132	0.119	0.120	0.181	0.352
**b) Admission test 2011 (N = 1702)**					
**Test part**	**Biology**	**Chemistry**	**Physics**	**Mathematics**	**Text comp.**
**Chemistry**	0.780				
**Physics**	0.614	0.668			
**Mathematics**	0.468	0.533	0.615		
**Text comp.**	0.447	0.401	0.397	0.459	
**SJT**	0.103	0.048	0.063	0.114	0.330
**c) Admission test 2012 (N = 1686)**					
**Test part**	**Biology**	**Chemistry**	**Physics**	**Mathematics**	**Text comp.**
**Chemistry**	0.788				
**Physics**	0.670	0.732			
**Mathematics**	0.495	0.588	0.615		
**Text comp.**	0.461	0.466	0.414	0.438	
**SJT**	0.193	0.177	0.147	0.143	0.351

*All correlation coefficients are significantly different from zero (P < 0.0001).

**Figure 1:**
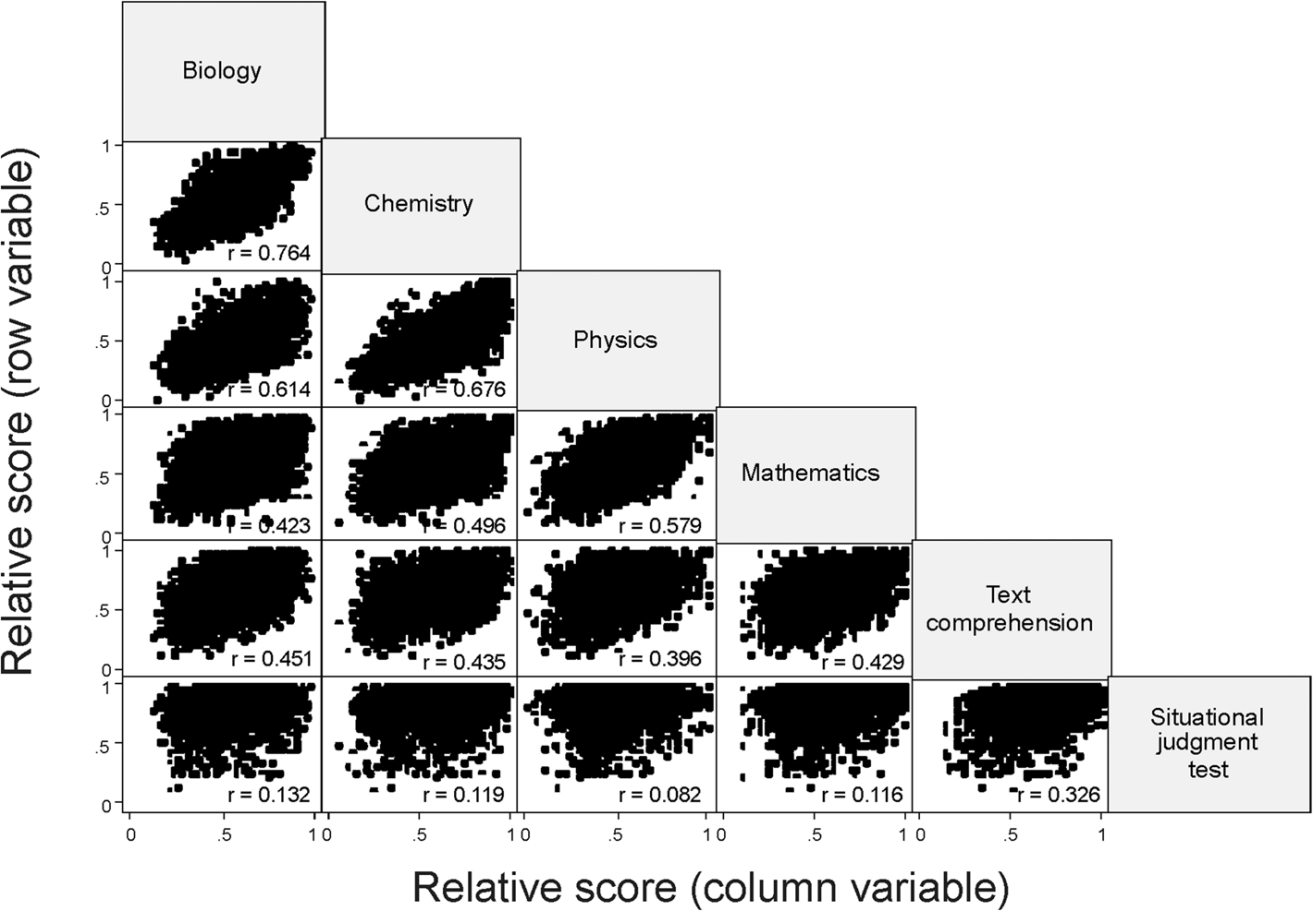
**Aggregated admission test results for three years.** Pairwise bivariate scatter plots of the relative scores of the various test parts, r, linear correlation coefficient.

Figure 1 visualizes the results aggregated over the three years: the strong correlation between biology and chemistry, and also the moderate correlations between the other test parts except SJT is obvious. The panels in the SJT row, however, show that the relative SJT scores are not nearly symmetrically distributed around a value of about 0.5; rather, most observations cluster in the high range above a relative score of 0.6, and apparently they do not depend on the relative score of the other test parts. This behavior of the relative SJT scores nicely reflects the fact that the SJT test part is the one with the least difficulty.

### Perceptions of the admission examination

**Figure 2:**
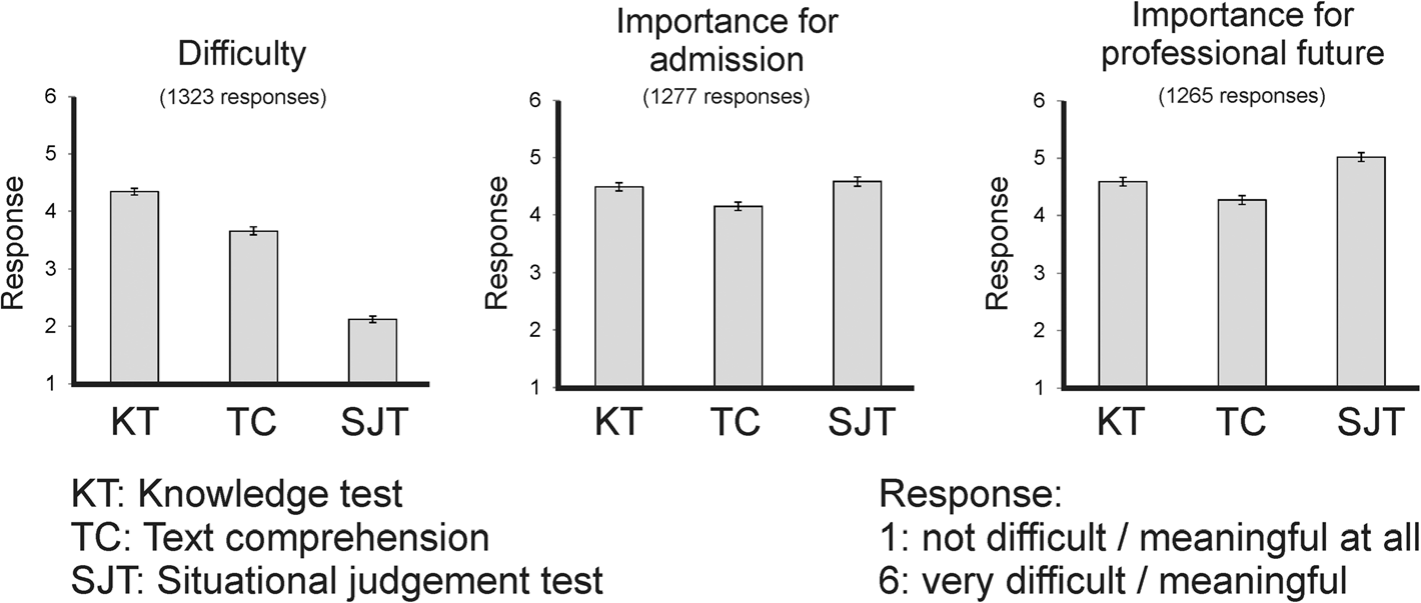
**Results of the evaluation of the admission procedure by the applicants.** The responses were on likert scales with six grades.

Figure 2 indicates that the SJT part is judged to present the least difficulty, while the knowledge test part is deemed to be the difficult part. Regarding the importance aspects of the test parts, the differences between the test parts were remarkably small; however, SJT was invariably regarded to be most important, both with respect to the admission procedure and the future professional life of the candidates. A similar rating by applicants was described by Lievens & Sackett (2006), among others: the written SJT as well as the video-based SJT were attested far more face-validity than the other parts of the admission exam [33].

## Conclusions

Inclusion of the SJT in an admission procedure for medical studies which previously was nearly exclusively based on scientific knowledge was demonstrated to be organizationally feasible in the presented manner. Moreover, the subjective responses of the applicants were quite positive, probably because of the felt relevance for the future study as well as profession. The lack of significant correlations between the other test parts and the SJT indicated that the spectrum of competencies tested was indeed broadened by inclusion of the SJT; a fact that seemed highly desirable in view of the overwhelming contribution of natural science knowledge to the admission test in the past.
